# P012 Hidden fractures: a case of bilateral lower limb Methotrexate Osteopathy

**DOI:** 10.1093/rap/rkag113.042

**Published:** 2026-11-03

**Authors:** Victoria Dunnett-Kane, Inês Soares

**Affiliations:** Wirral University Teaching Hospital NHS Foundation Trust, Upton, United Kingdom; Wirral University Teaching Hospital NHS Foundation Trust, Upton, United Kingdom

## Abstract

**Introduction:**

Methotrexate is a widely used therapy in rheumatological and inflammatory disease. Methotrexate osteopathy is a rare but increasingly recognised complication of treatment, characterised by pain, osteoporosis and atypical stress fractures. It is primarily observed in women with well controlled rheumatoid or psoriatic arthritis. Unlike typical osteoporotic fractures, methotrexate osteopathy affects weight-bearing bones of the lower limb, particularly the tibia and calcaneus. These present as transverse non-displaced fractures that are difficult to detect on plain x-ray. We present a case of suspected methotrexate osteopathy resulting in multiple bilateral lower limb insufficiency fractures that posed a diagnostic challenge.

**Case description:**

A 79 year old woman was under the care of our Rheumatology department with a diagnosis of psoriatic arthritis, which had been well controlled on methotrexate monotherapy since 2008. Her other past medical history was significant for skin psoriasis, osteopenia (neck of femur T score –1.9 in 2023) and osteoarthritis.

Starting in early 2024, the patient reported pain and swelling in her left foot and ankle. X-rays performed soon after showed degenerative changes only. She subsequently developed bilateral knee pain, for which she saw a private orthopaedic surgeon and was given a diagnosis of bilateral pes anserinus tendinopathy. Her right knee pain responded well to steroid injection. She then developed right foot and ankle pain and swelling. Of note, x-rays of the right knee, ankle and foot performed in early 2025 showed no evidence of fracture. Examination in June 2025 showed bilateral lower limb oedema with no clinical synovitis.

Due to persistent right ankle and foot pain impairing her mobility, an MRI was performed in October 2025, which revealed insufficiency fractures of the tibial plafond and body of the talus. Repeat DEXA showed advanced osteopenia with neck of femur T score –2.2. Due to ongoing left foot, ankle and knee pain, MRI left ankle and knee were performed in December 2025 showing multiple insufficiency fractures on the left side: of the medial femoral condyle, tibia, fibula head, and calcaneum. All fractures were managed conservatively by the orthopaedic team.

Due to concerns of methotrexate osteopathy, methotrexate therapy was discontinued and zoledronic acid was given for advanced osteopenia in the context of multiple fractures. At her last review in June 2026, her pain and mobility had significantly improved, although she was still experiencing right ankle pain.

**Discussion:**

This was an interesting case which prompts reflection on how we investigate mechanical type pain. Our patient first contacted the rheumatology helpline with foot and ankle pain around 18 months prior to her first MRI scan. However, it is difficult to know when her fractures occurred, and it is important to consider that there are many more common causes of pain in patients with an inflammatory arthritis. Our patient was reviewed by both rheumatology and orthopaedics who felt that her symptoms were more in keeping with alternative diagnoses (osteoarthritis and tendinopathy). Indeed, it is likely that there were multiple factors contributing to her lower limb pain over the two-year period. However, it does stress the importance of considering a broad range of differential diagnoses and avoiding diagnostic anchoring in patients with inflammatory arthritis.

Of note, our patient had x-rays of both ankles and knees prior to MRI scans. These revealed, at most, mild degenerative changes. In the context of persistent mechanical pain in a patient on methotrexate, we should be cognisant that x-rays may provide false reassurance and consider MRI as an early imaging modality.

This case also raises questions about optimal treatment strategy. Literature suggests that methotrexate cessation is the primary intervention. In our case, methotrexate was initially discontinued by the patient, who proactively researched the proposed diagnosis of methotrexate osteopathy. Certainly, literature suggests that discontinuation of methotrexate is associated with an absence of further fractures and better clinical outcomes. Antiresorptive therapy is also frequently used, although current evidence has yet to prove any additional benefit. Going forward, it is important to consider how we can offer the most effective treatment to patients with methotrexate osteopathy.

**Key learning points:**

1. Methotrexate osteopathy is a rare complication of methotrexate therapy but a critical differential diagnosis for lower limb pain. Unlike osteoporosis, fractures associated with methotrexate osteopathy primarily occur in the tibia and foot and are often bilateral, multiple and sometimes recurrent.

2. Plain x-ray cannot exclude fractures in methotrexate osteopathy and MRI should be performed if there is high clinical suspicion.

3. The main treatment strategy is methotrexate discontinuation, which often results in significant improvement in symptoms and favourable outcomes. Beyond this, osteoporosis management is frequently used, although its effectiveness is not yet clear. Due to the complexity of managing this rare condition, it is important that these patients are managed in a multi-disciplinary setting.

